# Comparison of Self-Reported Telephone Interviewing and Web-Based Survey Responses: Findings From the Second Australian Young and Well National Survey

**DOI:** 10.2196/mental.8222

**Published:** 2017-09-26

**Authors:** Alyssa C Milton, Louise A Ellis, Tracey A Davenport, Jane M Burns, Ian B Hickie

**Affiliations:** ^1^ Brain and Mind Centre University of Sydney Camperdown Australia; ^2^ Australian Institute of Health Innovation Macquarie University Sydney Australia

**Keywords:** survey methods, youth, mental health, online behaviors, information disclosure

## Abstract

**Background:**

Web-based self-report surveying has increased in popularity, as it can rapidly yield large samples at a low cost. Despite this increase in popularity, in the area of youth mental health, there is a distinct lack of research comparing the results of Web-based self-report surveys with the more traditional and widely accepted computer-assisted telephone interviewing (CATI).

**Objective:**

The Second Australian Young and Well National Survey 2014 sought to compare differences in respondent response patterns using matched items on CATI versus a Web-based self-report survey. The aim of this study was to examine whether responses varied as a result of item sensitivity, that is, the item’s susceptibility to exaggeration on underreporting and to assess whether certain subgroups demonstrated this effect to a greater extent.

**Methods:**

A subsample of young people aged 16 to 25 years (N=101), recruited through the Second Australian Young and Well National Survey 2014, completed the identical items on two occasions: via CATI and via Web-based self-report survey. Respondents also rated perceived item sensitivity.

**Results:**

When comparing CATI with the Web-based self-report survey, a Wilcoxon signed-rank analysis showed that respondents answered 14 of the 42 matched items in a significantly different way. Significant variation in responses (CATI vs Web-based) was more frequent if the item was also rated by the respondents as *highly sensitive* in nature. Specifically, 63% (5/8) of the *high sensitivity* items, 43% (3/7) of the *neutral sensitivity* items, and 0% (0/4) of the *low sensitivity* items were answered in a significantly different manner by respondents when comparing their matched CATI and Web-based question responses. The items that were perceived as *highly sensitive* by respondents and demonstrated response variability included the following: sexting activities, body image concerns, experience of diagnosis, and suicidal ideation. For *high sensitivity* items, a regression analysis showed respondents who were male (beta=−.19, *P*=.048) or who were not in employment, education, or training (NEET; beta=−.32, *P*=.001) were significantly more likely to provide different responses on matched items when responding in the CATI as compared with the Web-based self-report survey. The Web-based self-report survey, however, demonstrated some evidence of avidity and attrition bias.

**Conclusions:**

Compared with CATI, Web-based self-report surveys are highly cost-effective and had higher rates of self-disclosure on sensitive items, particularly for respondents who identify as male and NEET. A drawback to Web-based surveying methodologies, however, includes the limited control over avidity bias and the greater incidence of attrition bias. These findings have important implications for further development of survey methods in the area of health and well-being, especially when considering research topics (in this case diagnosis, suicidal ideation, sexting, and body image) and groups that are being recruited (young people, males, and NEET).

## Introduction

Over the past decade, the Australian government and nongovernment organizations have invested heavily in computer-assisted telephone interviewing (CATI) and face-to-face interview methodologies [1-4]. Of these, CATI has proved more popular than face-to-face surveys as it has greater cost-effectiveness, has good geographical coverage without the need of travel, and maintains a personal interaction between the interviewer and the survey respondent, while also offering random digit dialing (RDD) for sample selection. However, in more recent years, changes in technology have resulted in some new challenges to using telephone surveying [5]. Landline telephone use has decreased because of mobile phone popularity. This has hindered sample stratification, which was traditionally enabled by an association between landlines and geographic locations. Decreasing CATI response rates and increased sampling bias have also been attributed to the use of certain technologies including do not call registers and the use of voicemail and caller identification [6]. With the rapid uptake of the Internet over the past decade, facilitated by better connectivity (eg, Wi-Fi and national fiber optics networks), Web-based self-report surveying has become increasingly popular for its potential to efficiently yield much larger samples at a much lower cost [7]. Despite this popularity, research comparing respondent answers in youth-focused mental health surveys when using CATI versus Web-based self-report surveys is limited.

There are some subgroups of the population that may find Web-based self-report surveys particularly advantageous. Research has suggested that young people are more comfortable using the Internet than other subsamples of the population [8]. The Internet is also widely accessible to nearly all young people [1,9]. Since 2008, our research group has focused on national surveys relating to mental health and technology use of young people. These have been carried out using both CATI [1,10] and Web-based self-report surveys [1,10-12]. Results from the First Australian Young and Well National Survey [1] showed that young people’s responses differed based on methodologies (CATI vs Web-based self-report survey). For example, there was a higher proportion of young people reporting psychological distress online compared with CATI (59% vs 21% *high* to *very high* distress).

Another subsample that Web-based self-report surveys may benefit comprises men and boys. Research has suggested that males have poorer mental health knowledge and higher mental health stigma than females [8], and they are also more reluctant to disclose sensitive mental health information [13]. Alongside further investigation into general response differences when comparing CATI with Web-based self-report surveys, gender differences in responding warrants further investigation. This line of inquiry follows our previous research into young men and their technology use, mental health help-seeking, and stigma [1,11,12,14].

There are other advantages and disadvantages to collecting self-report data online versus CATI. For example, particular challenges arise when survey questions contain information that is considered sensitive in nature. According to Tourangeau and Yan [15], survey questions can be labeled as “sensitive” if respondents perceive them as intrusive or an invasion of privacy, they raise fears about the potential repercussions of disclosing the information, or if they trigger social desirability concerns. Examples of sensitive topics that appear in the literature include illicit drug use, abortion and, sexual behavior [15].

There is evidence to suggest that with the presence of an interviewer, a social desirability effect occurs, whereby respondents minimize more unpleasant disclosures to maximize social acceptability and respectability [16]. Responses to more sensitive items may be reported at a higher rate online than via the telephone. For example, higher levels of alcohol consumption have been reported by college students answering online compared with those responding via the telephone [17]. More importantly, most prior research in this domain has relied on the researchers’ judgments about which items are sensitive (consider the study by Kreuter et al [18] as an exception). Therefore, what is considered sensitive from respondents’ perspective and how this influences their responses requires further research.

For the Second Australian Young and Well National Survey 2014 (Second National Survey, 2014), we sought to more thoroughly compare a CATI with a Web-based-self-report survey. This subsidiary study had three main aims, which were to assess whether (1) there were within-subject response differences between the CATI and the Web-based self-report survey; (2) sensitive items demonstrated greater variability across the different methodologies (CATI vs Web-based survey) compared with nonsensitive items; and (3) particular respondent subsamples demonstrated greater variability in their responses between methodologies based on the sensitivity of the items.

## Methods

### Design and Sample

This research was a subsidiary study that formed part of a larger national research project in Australia, the Second National Survey (2014). A within-subjects design was used in this substudy to assess response differences of respondents using matched items at two time points delivered via two different methodologies (CATI vs Web-based self-report survey).

To participate in this substudy, respondents were recruited from the larger Second National Survey (2014). Cross-sectional CATI using RDD to fixed landlines and mobile phones (N=1400) and a Web-based self-report survey (N=2416) were used to explore young people’s experiences of mental health and well-being and their use of information and communication technology. The sampling and methods used in the CATI and the Web-based self-report survey for the larger Second National Survey (2014), followed the First Young and Well National Survey 2012, which have been described in further detail elsewhere [1].

**Figure 1:**
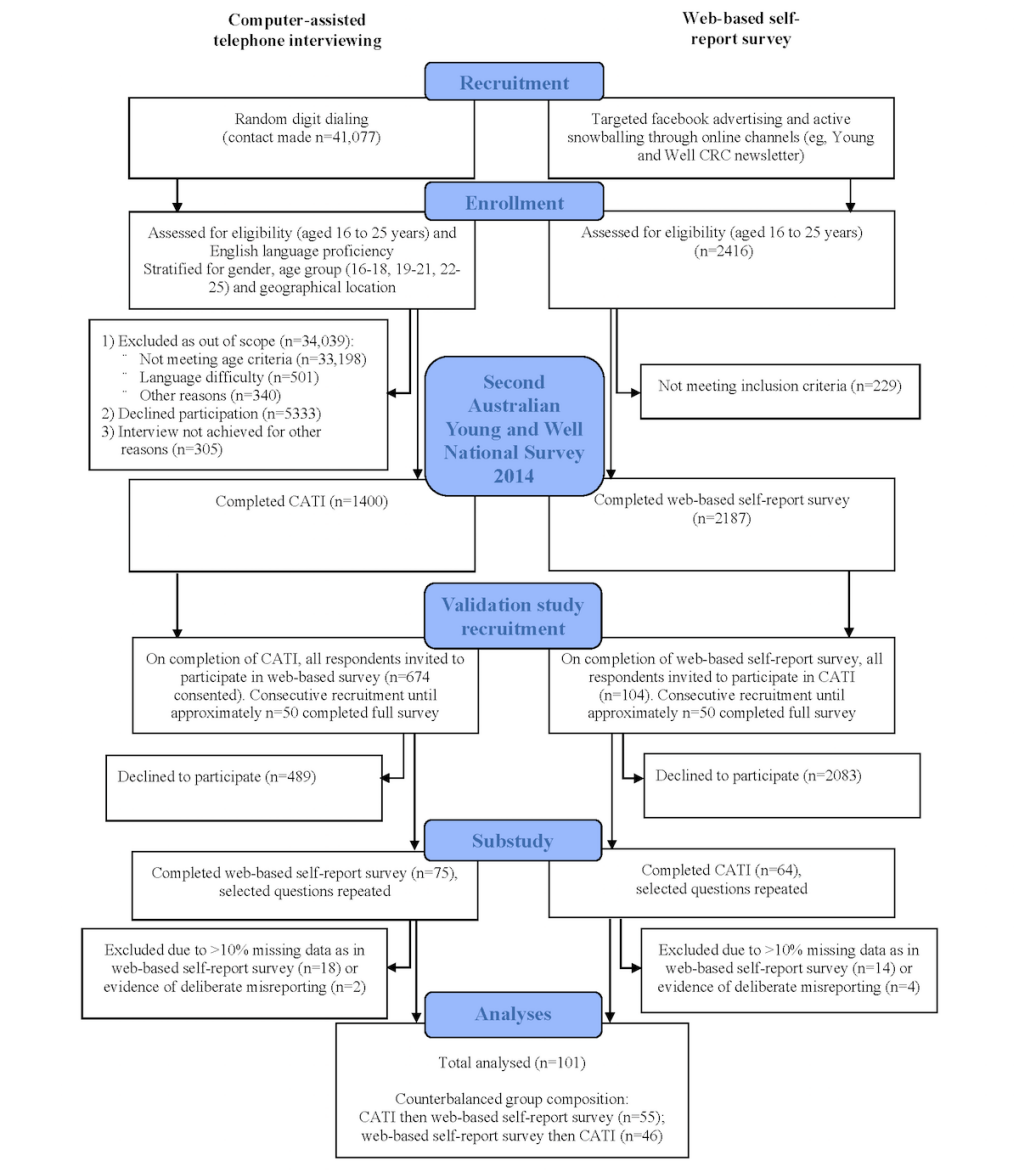
Study flow.

At the end of both the CATI and the Web-based self-report survey, respondents were asked to provide an email account if they would be willing to be contacted again for research purposes on the same topic. Second National Survey (2014) CATI respondents who gave consent to be contacted again (N=674) were sent an email link to a smaller Web-based self-report survey with specific items drawn from the Second National Survey (2014) selected for this substudy. The Second National Survey (2014) Web-based respondents who gave consent to be contacted again and provided their telephone details (N=104) were contacted via telephone and were asked the same matched items selected for this substudy. Hence, respondents in this substudy completed the same set of questions on two occasions (via CATI and Web-based self-report survey), and the order of survey completion was counterbalanced. Consecutive recruitment took place until 100 useable cases were completed by respondents at these two time points (CATI and Web-based self-report survey). Study flow is shown in Figure 1. This study received ethics approval from The University of Sydney Human Research Ethics Committee (Protocol No. 2014/741).

### Items

The initial Second National Survey (2014) was administered using both a CATI and a Web-based self-report survey and included up to 81 questions, depending on the skip pattern. Questions included demographics, general health and well-being, mental health, health perceptions of Australian youth, use of the Internet, online and communication risks (eg, digital abuse such as bullying and sexting), happiness and resilience, social networking and relationships, as well as use of mobile phones, apps, and social media. To address the primary aim outlined in this substudy, 19 questions (42 items and subitems in total) were selected to be administered using both the CATI and the Web-based self-report survey to the same respondent on two occasions. These items were purposively selected to provide a range of potentially sensitive and nonsensitive items that were both personal and nonpersonal in nature. Selected items are presented in Multimedia Appendix 1. The Second National Survey (2014) results from both the CATI and the Web-based self-report survey full samples for these selected matched items are included in Multimedia Appendix 2 as frequency statistics.

To address the secondary and tertiary aims relating to understanding item sensitivity, the final item of the survey, administered at the second time point only, asked respondents to rate the sensitivity of some of the earlier items. Similar to Kreuter and colleagues [18], this question read: “Questions sometimes have different effects on people. We’d like your opinions about some of the questions in this survey. Please indicate the degree to which you think each of the following items might make people falsely report or exaggerate their answers?” Respondents rated each sensitivity question on a 5-point Likert scale of 1= *strongly disagree* to 5= *strongly agree*.

### Statistical Analysis

Data were analyzed using IBM’s Statistical Package for the Social Sciences (SPSS) version 21.0. A Wilcoxon signed-rank test was conducted to address the primary aim of the study. This analysis was used to determine whether respondents’ median scores differed for each repeated matched item at the two measurement points (CATI and Web-based self-report survey). This test was used, as the sample could not be assumed to be normally distributed. There was sufficient sample size (a priori minimum N=94, actual sample achieved N=101) as determined by G*Power 3.1 (a priori Cronbach alpha=.05, minimum effect size=0.3, power=0.8; [19]). Multiple response categorical items were collapsed to meet assumptions of the Wilcoxon signed-rank test that the dependent measurements were at least of ordinal scale (which includes dichotomous measures). This included collapsing: Q3, main educational and vocational activity (not in employment, education, or training [NEET] vs in employment, education, or training [EET]); Q4, highest level of education (tertiary vs nontertiary); and, Q12 and Q13, weekday and weekend Internet use (regular hours use vs late night use [11pm to 5am]). All Likert scale items and dichotomous categorical items were not transformed at this point. Missing values (“Don’t know” or “Refused”) were excluded from analysis, except where stated in Table 1, to meet the required ordinal scale assumption.

To address the secondary aim of the study, a number of analyses assessing item sensitivity were performed. First, the sensitivity items (adapted from Krueter et al [18]) were collected at the second survey time point using two different methodologies (CATI and Web-based self-report survey). Thus, a Mann-Whitney *U* test was used to examine if there were any significant differences in responses between the two methodologies for each sensitivity item. Following this, items were aggregated by their median score into *high sensitivity*, *neutral sensitivity*, and *low sensitivity* groups. Each item that received a median score of 4 was allocated to the *high sensitivity* group, as the majority of respondents “agreed” that people might falsely report or exaggerate their answers. An item with a median score of 3 indicated *neutral sensitivity,* as the majority of respondents “neither agreed nor disagreed” that people might falsely report or exaggerate their answers. Finally, an item that received a median score of 2 was categorized into the *low sensitivity* group, as the majority of respondents “disagreed” that people might falsely report or exaggerate their answers. Within these three established groups (*low*, *neutral*, and *high*), a difference score between the CATI and the Web-based self-report survey was calculated. This was carried out by determining whether the respondent provided the same response using the two methodologies (difference score=0) or provided a different response (difference score=1) for each identical item collapsed onto dichotomous measures.

To address the tertiary aim of the study, each item’s difference score was then aggregated into a total difference score for each sensitivity group (*low*, *neutral*, and *high*), and the mean score was taken as each sensitivity group had varying numbers of items. Subsequent regression analyses were used to examine the association between demographic or biographic items and the total mean differences score for the sensitivity groups (*low*, *neutral*, and *high*). There was sufficient sample size (a priori minimum N=89, actual sample achieved N=101) calculated using G*Power 3.1 ([19]; a priori Cronbach alpha=.05, minimum effect size=0.2, power=0.9, predictors=5). Five demographic or biographic predictors that met regression model assumptions of linearity, homoscedasticity, independence, and normality (evaluated using standard residuals-based diagnostic procedures) included gender (males vs females), age, educational attainment (tertiary vs nontertiary), main vocational and educational activity (NEET vs EET), and the order of survey administration (CATI first vs Web-based self-report survey first).

## Results

### Sample

A total of 139 respondents completed both the CATI and Web-based self-report survey. Of these, 32 cases (23.0%, 32/139) were discarded as too much data were missing from one administration (>10% of data missing on one administration in the Web-based sample), and a further 6 cases (4.3%, 6/139) were discarded because of obvious misreporting (ie, unreasonable values for some or all data fields). All missing data and misreporting cases were from the Web-based self-report survey. Of the discarded cases, approximately half were female (55%, 21/38). Overall, 101 respondents provided sufficient data for analysis. Of these remaining cases, 61 (60.4%) were female, 40 (39.6%) were male, 55 (54.5%) completed the CATI before the Web-based self-report survey, and 46 (45.5%) completed the Web-based self-report survey before the CATI.

### CATI Versus Web-Based Response Differences (Primary Aim Findings)

Results from the Wilcoxon signed-rank test by matched item (see Multimedia Appendix 1) showed that 14 of the matched items demonstrated significant median differences between the CATI and Web-based self-report survey. The vast majority of these matched items (13/14, 93%) that demonstrated significant differences were consistently ranked in the same direction. Specifically, respondents endorsed the item when asked via CATI less frequently than when answering online. These items included main educational and vocational activity (NEET: CATI item endorsement=5.0% vs Web-based self-report survey=17.8%; *P­*=.002), experience of diagnosis (mental health or behavioral: CATI item endorsement=25.0% vs Web-based self-report survey=31.6%; *P*=.03), suicidal ideation (thought of taking own life: CATI item endorsement=11.1% vs Web-based self-report survey=18.4%; *P*=.02), multiple sexting activity responses, searching for information relating to a health problem (mental health or substance use problem: CATI item endorsement=61.4% vs Web-based self-report survey=74.3%; *P­*=.002), help-seeking waiting periods (less than 4 weeks: CATI item endorsement=66.3% vs Web-based self-report survey=72.0%; *P­*=.003), and body image (CATI item endorsement=34.7% vs Web-based self-report survey=65.3%; *P*<.001). Only one item “Do you think cyberbullying is a serious problem for young people?” had higher respondent endorsement in the CATI than Web-based self-report survey (CATI item endorsement=93.1% vs Web-based self-report survey=80.6%; *P*=.02).

### Item Sensitivity (Secondary Aim Findings)

The Mann-Whitney test determined that there were no differences between CATI and Web-based self-report respondent ratings of sensitivity items. Table 1 shows the perceived sensitivity of each item, as rated by the respondent, and their subsequent sensitivity group allocation based on median scores. All items that demonstrated significant differences in the initial Wilcoxon signed-rank test were found to be in the *high sensitivity* or *neutral sensitivity* group, with no differences found in the *low sensitivity* group. A greater proportion of *high sensitivity* domains (5/8, 63%) demonstrated significant differences between surveying methodologies in the Wilcoxon signed-rank test compared with the *neutral sensitivity* (3/7, 43%) and *low sensitivity* (0/4, 0%) domains.

### Item Sensitivity by Subgroup (Tertiary Aim Findings)

The linear regression models for each of the sensitivity groupings (*low*, *neutral*, and *high*) are presented in Table 2. For the *low sensitivity* group, none of the demographic or biographic variables were significant in explaining the regression model’s variance (*F*_5,95_=0.45, *P*=.81, *R*^2^_adj_=−.03). For the *neutral sensitivity* group, 8% of the variance was significantly accounted for in the regression model (*F*_5,95_=2.8, *P*=.02, *R*^2^_adj_=.08). Two variables significantly explained the variance, which included age (beta=.28, *P*=.03) and EET status (beta=−.26, *P*=.01). Those who were older or NEET had higher total mean difference scores for *neutral sensitivity* items. That is, they more frequently reported differently online than they did in the CATI for *neutral sensitivity* items. For the *high sensitivity* group, 14% of the variance was significantly accounted for in the regression model (*F*_5,95_=4.3, *P*=.001, *R*^2^_adj_=.14). Two variables significantly explained the variance, which included sex (beta=−.19, *P*=.048) and EET status (beta=−.32, *P*=.001). Those who were male or NEET had higher total mean difference scores for *high sensitivity* items. That is, they more frequently reported differently online than they did in the CATI when answering *high sensitivity* items.

For males, items with the highest difference in item responses were Q20ii, whether body image is an issue that concerns them personally (CATI: 5/39, 13% vs Web-based self-report survey: 18/40, 45%); Q18v, whether they have seen other people perform acts of a sexual nature on their mobile phone, smartphone, or the Internet (CATI: 11/40, 28% vs Web-based self-report survey: 22/40, 55%); and Q19i, whether they had sent someone a sexual message on their mobile phone, smartphone, or the Internet (CATI: 13/40, 33% vs Web-based self-report survey: 21/40, 53%).

**Table 1:** Perceived sensitivity of survey items.

Survey item^a^	Perceived sensitivity			Sensitivity grouping
	N	Median	Mean (standard deviation)	
16ii. In the past 12 months, how often have you cyber-bullied someone?	93	4	4.01 (1.03)	High
19. In the past 12 months, have you done these things (sexting activities) on your mobile, smartphone, or the Internet?	93	4	3.94 (1.08)	High^b^
5. How would you rate your overall mental health in the past 4 weeks?	90	4	3.83 (0.82)	High
18. In the past 12 months, have you had any of these things (sexting activities) happen to on your mobile, smartphone, or the Internet?	93	4	3.75 (1.06)	High^b^
7. In the past 12 months have you ever thought about taking your own life?	91	4	3.74 (1.07)	High^b^
6. Have you ever been diagnosed with a mental health or behavioral problem?	91	4	3.74 (0.85)	High^b^
16ii. In the past 12 months, how often have you been cyber-bullied?	94	4	3.51 (1.12)	High
2. Do any of the following issues concern you personally (eg, alcohol, body image, and depression)?	93	4	3.44 (1.07)	High^b^
4. What is your highest level of education?	93	3	3.00 (1.12)	Neutral
14. Have you ever used the Internet to find information for a mental health, alcohol, or substance use problem?	92	3	2.98 (1.15)	Neutral^b^
17. Do you think sexting is a serious problem for young people your age?	91	3	2.91 (1.21)	Neutral
9. Would you know where to get help if you, or someone you knew, was feeling suicidal?	88	3	2.88 (1.19)	Neutral
8. How long do you think a mental health or behavioral problem needs to be present before a young person should seek help?	88	3	2.76 (1.09)	Neutral^b^
3. Main current activity	93	3	2.69 (1.17)	Neutral^b^
15. Do you think cyberbullying is a serious problem for young people?	93	3	2.63 (1.20)	Neutral
11. How often do you use the Internet?	98	2	2.66 (1.39)	Low
12. When are you most active online on a normal weekday or workday?	96	2	2.35 (1.27)	Low
13. When are you most active online on a normal weekend or nonworkday?	97	2	2.34 (1.22)	Low
1. Do you use the Internet?	97	2	2.25 (1.30)	Low

^a^Ordered from most sensitive to least sensitive items. Sensitivity grouping is based on median score.

^b^Denotes items that demonstrated at least some significant difference in respondent answers when using the CATI and the online self-report survey (as measured by the Wilcoxon singed-rank test).

**Table 2:** Multiple regression models for total mean difference scores in high sensitivity, neutral sensitivity, and low sensitivity groups.

Variable		*t*	*P* value	Beta	95% CI	*F*	*df*^a^	*P* value	Adjusted *R*^2^
**High sensitivity**						4.30	5,95	.001	.14
	Sex	−2.00	.048	−.19	−0.10 to 0.00				
	Age	−1.14	.26	−.14	−0.02 to 0.05				
	EET^b^ status	−3.29	.001	−.32	−0.16 to −0.04				
	Educational attainment	0.80	.42	.10	−0.04 to 0.09				
	Order of survey completion	−1.41	.16	−.13	−0.08 to 0.01				
**Neutral sensitivity**						2.81	5,95	.02	.08
	Sex	1.32	.19	.13	−0.02 to 0.08				
	Age	2.17	.03	.28	0.00 to 0.02				
	EET status	−2.62	.01	−.26	−0.15 to −0.02				
	Educational attainment	−1.90	.06	−.25	−0.13 to 0.00				
	Order of survey completion	1.26	.21	.12	−0.02 to 0.08				
**Low sensitivity**						0.45	5,95	.81	−.03
	Sex	0.59	.56	.06	−0.03 to 0.05				
	Age	0.32	.75	.04	−0.01 to 0.01				
	EET status	0.51	.61	.06	−0.04 to 0.07				
	Educational attainment	−0.06	.96	−.01	−0.06 to 0.05				
	Order of survey completion	1.03	.31	.11	−0.02 to 0.06				

^a^df: degrees of freedom.

^b^EET: education, employment, or training.

## Discussion

### Principal Findings

The key findings of this research demonstrated that significant variation in responses (CATI vs Web-based) was more frequent if the item was also rated by the respondents as *highly sensitive* in nature. For these *high sensitivity* items, a regression analysis showed that male and NEET respondents were significantly more likely to provide different responses on matched items when responding in the CATI as compared with the Web-based self-report survey.

The primary aim of this study was to determine whether differences in survey responses arose using a within-subjects design that delivered the survey via two distinct methodologies; CATI versus Web-based self-report survey. Of the total 42 matching demographic, mental health, and well-being questions asked at the two counterbalanced survey time points, 14 (33%) demonstrated significant differences in respondent answers. These findings suggest that CATI and Web-based surveying approaches do not always yield corresponding results for the same individual surveyed. Importantly, the overall trend was that the Web-based self-report survey resulted in higher levels of disclosure or item endorsement.

The secondary aim explored potential reasons for these differences in respondent answers by examining item sensitivity. Respondent-rated *high sensitivity* items in this study included cyberbullying behavior, sexting activities, overall mental health, suicidal ideation, mental health diagnosis, and personally concerning issues such as body image. Of these, sexting activity, suicidal ideation, experiencing a mental health diagnosis, and body image concerns were endorsed significantly more frequently in the Web-based self-report survey compared with the CATI.

Previous studies have found that respondents report more socially undesirable sexual behavior in self-administered questionnaires than interviewer-administrated surveys [20,21]. Although related to these previous studies, no known research has compared Web-based self-report surveys with CATI for sexting behavior. This finding is important as survey-based research into sexting activities often cites social desirability bias as a key limitation to their findings [22,23]. Moving to Web-based self-report survey platforms may help to provide a more accurate understanding of the sexting landscape.

Similarly, no known research has compared CATI and Web-based self-report surveying methodologies when looking at questions relating to body image concerns. Our research found that, for males, this was the most underreported item in the CATI when compared with the self-report Web-based survey. Research has shown that for men in particular, body image is a difficult topic to discuss, especially when disclosing their insecurities, as they may be inexperienced at discussing how they feel about the way that they look [24]. Compared with surveys with an interviewer present, Web-based reporting may allow people, especially men, to open up more freely about any body image concerns they are experiencing.

When compared with interviewer-administered surveys, self-report computer-based surveying has been found to increase respondents’ reports of mental health symptoms [25]. Contrary results, however, have been reported in other studies on mental health symptomology [26], and other research has found no difference between the rates of reporting depression symptoms [27]. There was no difference in overall mental health ratings in this substudy when comparing surveying methodologies despite this item being rated as a *highly sensitive* item. Respondents did, however, report significantly higher rates of suicidal ideation and diagnosis in the self-report Web-based survey. One reason for this difference may be attributed to both suicidal ideation and the experience of a mental health diagnosis being harder to disclose to an interviewer than a person’s overall mental health. This may be compounded by the response options provided, in that overall mental health provided a range of response options on a 5-point Likert scale (ranging from very bad to very good), whereas suicidal ideation and mental health diagnosis elicited binary responses of “yes” or “no.”

Overall, a larger proportion of items deemed by respondents as more sensitive had greater susceptibility to variance, as compared with those that were rated as having *low sensitivity*. With the exception of one item, all items demonstrating significant differences were more frequently endorsed using the Web-based self-report survey than the CATI. To some extent, this finding supports the research highlighting that more personal, unpleasant, or self-stigmatizing disclosures are minimized in the presence of an interviewer and are more frequently endorsed online (eg, [16,17,28]). Interestingly, the one item endorsed significantly more frequently in the CATI related to cyberbullying being a serious concern for young people. Although this may simply be due to the question being more general and not specific to the individual, this may also be due to the effect of social desirability; a respondent endorsing that cyberbullying is an issue may be associated with a belief that greater social approval will be provided by the interviewer who is researching the topic.

These findings are highly relevant to youth mental health and well-being surveys, as such surveys typically involve sensitive questions, and research has suggested that young people have a fear of stigma relating to mental health problems, as well as increased concerns regarding confidentiality [29]. Web-based surveys may help minimize these concerns. It is also important to consider how young people use technology and the influence this use may have on self-disclosure of sensitive information. Today, young people are known to disclose significant amounts of sensitive information through social networking and texting. For example, a recent study suggests that adolescents disclose more on social media and use privacy settings less than adults [30]. Given their propensity to use social media and online channels to discuss sensitive information with others, young people arguably generalize these behaviors to other online scenarios such as responding to surveys. Thus, current trends in use of these mediums for self-disclosure may be instilling in this generation a greater willingness to disclose in online formats [31]. Web-based self-report surveys may pose challenges with some other populations, including those less familiar with technology, those with language or literacy issues, and those less likely to have readily available and affordable Internet access (eg, the elderly and those with a lower socioeconomic status). The question of Internet access and acceptability, however, does not appear to be an issue for young Australians, who are native to technology in their daily lives, with 99% reporting daily Internet use in our larger Second National Survey (2014) in both the CATI and the Web-based self-report survey.

The tertiary aim of the study was to examine the impact item sensitivity had on specific subgroups. Results showed that those who were male and those who were NEET were more susceptible to variance in disclosure of highly sensitive items. These groups exhibited a higher endorsement of items when answering a Web-based self-report survey. Research comparing gender differences in responses with sensitive items using Web-based self-report and CATI surveying methodologies is lacking. However, Web-based survey research has reported that in situations where privacy is perceived to be greater, men have significantly higher disclosure rates when asked sensitive questions, whereas women maintain a stable disclosure rate irrespective of the privacy condition [32]. Thus, in this substudy, the anonymity of the Web-based self-report survey may be seen as influencing men’s willingness to disclose more sensitive information. In general, research suggests that men are reluctant to disclose sensitive mental health information [13]. This may be attributed to the greater levels of mental health stigma men experience. For example, a 2015 systematic review [33] reported that compared with women, men were disproportionately deterred to seek help for their mental health because of stigma, with disclosure concerns the most commonly reported stigma barrier. Similarly, in depression assessments, males tend to underreport symptoms of depression that should require medical attention [34]. Overall, the findings of increased levels of disclosure for males in this substudy suggest that Web-based self-report surveys may be useful to assist males to disclose more openly.

Item sensitivity ratings explain at least some variability across survey methodologies; however, sensitivity ratings do not explain all variability between the two methodologies. Some matched items (such as a respondent’s mental health rating over the past 12 months) were not significantly different between the CATI and the Web-based self-report survey within this substudy, although differences were expected. This particular item showed considerable differences in the larger Second National Survey (2014) samples, with CATI respondents reporting better overall mental health (eg, the CATI median rating was “good” with 39.6% of respondents reporting this score, whereas the Web-based self-report survey median rating was “moderate,” representing 32.4% of respondents). As no differences in these items were found in this substudy, the disparities found in the full Second National Survey (2014) may be attributed in some capacity to recruitment methods when sampling online. In particular, avidity bias may be involved as those with a greater interest in, or experience with, a survey topic are more likely to respond [35]. In the Web-based self-report Second National Survey (2014) sample, it may be that people with a lived experience of mental health problems were more likely to want to participate in a study focused on health and well-being, which explains the higher distress levels compared with the CATI.

In online recruitment, there is no social desirability pressure (albeit unspoken, unintentional, and subconscious) from an interviewer to initially take part in a study, unlike when contacted by the CATI through RDD. Interviewer presence may also explain the attrition bias that arose in the Web-based self-report survey sample. Specifically, in this substudy, all cases that were excluded because of missing data arose from Web-based surveying, that is, respondents are far less likely to terminate a CATI.

### Strengths and Limitations

A key strength of this study was that respondents completed matched questions via the CATI and the Web-based survey. The wording of the questions and response categories were as identical as possible for each methodology to ensure consistency and comparability of responses. A further strength was the inclusion of ratings of item sensitivity at the end of the survey. This is important as sensitivity groupings were therefore based on the respondents’ perceptions rather than researchers’ assumptions. Despite these strengths, this was a comparative study of CATI vs Web-based self-report survey responses without the possibility of external validation with some objective criterion. We are essentially interpreting the results according to the “more-is-better” assumption for socially undesirable behavior and the “less-is-better” assumption for socially desirable behavior, respectively [28].

In terms of sampling, the strength of the study lay in the initial random sampling of respondents. A major limitation, however, was that the respondents then volunteered to take part in the second survey. As described above, this may result in avidity bias. Future studies should consider embedding random sampling across both survey time points into the study design. Furthermore, for a fine-grained comparison, an additional two control samples could have been included in the design of the study. In this future design, participants would complete the identical questions on two occasions but use the same surveying methodology (CATI vs CATI and Web-based vs Web-based).

### Conclusions

The CATI, although a popular methodology, may be susceptible to underreporting when eliciting sensitive demographic or biographic, mental health, and well-being information from young people. Therefore, there may be some benefits to using Internet-based self-report surveys in research with young people when collecting data on sensitive issues, especially those related to body image concerns, suicidal ideation, and viewing or receiving sexual content online. However, there are also disadvantages in using Web-based surveys, which are important to take into consideration, particularly because of the concerns around nonrepresentative sampling due to avidity and attrition bias. Overall, researchers must consider the best fit in survey format with the population being studied [23]. In the case of researching sensitive mental health and well-being questions with young people (especially males and those who are NEET), a Web-based self-report survey may facilitate improved rates of self-disclosure.

## Appendix

Multimedia Appendix 1 Mean rank differences between identical items administered using CATI and an online self-report survey.

Multimedia Appendix 2 Second Australian Young and Well National Survey 2014 results and sub-study results.

## Abbreviations

CATI: computer-assisted telephone interviewing
EET: employment, education, and training
NEET: not in employment, education, or training
RDD: random digit dialing
SPSS: Statistical Package for the Social Sciences
