# Supplementary material for: Comparison of Self-Reported Telephone Interviewing and Web-Based Survey Responses: Findings From the Second Australian Young and Well National Survey
Source: JMIR Ment Health. 2017 Sep 26;4(3):e37. doi: 10.2196/mental.8222 (PMC5635234; doi:10.2196/mental.8222)
Supplement: Multimedia Appendix 1 [file mental_v4i3e37_app1.pdf]

Multimedia Appendix 1.

Mean rank differences between identical items administered using CATI and web-based self-report survey

**Survey item**	**CATI item endorsement (%[a])**	**Online self-report survey item endorsement (%[a])**	**Wilcoxon signed-rank test ranking**	**n**	**z**	***P***
**1. Sex (female)**	6	6				
			Negative Ranks	0	.00	1.00
			Positive Ranks	0		
			Ties	101		
**2. Age[b]**	2 (3)	2 (3)				
			Negative Ranks	0	.00	1.00
			Positive Ranks	0		
			Ties	101		
**3. Main educational/ vocational activity (NEET)**	5	18				
			Negative Ranks	15	-3.15	.002
			Positive Ranks	2		
			Ties	84		
**4. Highest level of education (tertiary)**	54	52				
			Negative Ranks	6	-.63	.53
			Positive Ranks	4		
			Ties	91		
**5. How would you rate your overall mental health in the past four weeks?**						
very good	66	24				
good	38	40				
moderate	27	27				
bad	4	8				
very bad	3	2				
			Negative Ranks	12	-1.40	.15
			Positive Ranks	25		
			Ties	64		
**6. Have you ever been diagnosed with**	25	32				

**a mental health or behavioural problem? (yes)**						
			Negative Ranks	1	-2.12	.03
			Positive Ranks	7		
			Ties	89		
**7i. In the past 12 months have you ever felt that life is hardly worth living? (yes)**	23	27				
			Negative Ranks	3	-1.27	.21
			Positive Ranks	7		
			Ties	85		
**7ii. In the past 12 months have you ever thought about taking your own life? (yes)**	11	18				
			Negative Ranks	1	-2.33	.02
			Positive Ranks	8		
			Ties	88		
**8. How long do you think a mental health or behavioural problem needs to be present before a young person should seek help? (< 4 weeks)**	66	72				
			Negative Ranks	22	-2.97	.003
			Positive Ranks	7		
			Ties	71		
**9. Would you know where to get help if you, or someone you knew, was feeling suicidal? (agree/ strongly agree)**	83	81				
			Negative Ranks	21	-1.62	.10
			Positive Ranks	12		
			Ties	65		
**1. Do you use the internet? (yes)**	10	10				

			Negative Ranks	0	.00	1.00
			Positive Ranks	0		
			Ties	101		
**11. How often do you use the Internet? (everyday/ almost everyday)**	10	10				
			Negative Ranks	0	.00	1.00
			Positive Ranks	0		
			Ties	101		
**12. When are you most active online on a normal weekday/ workday? (regular daytime/ evening use)**	97	97				
			Negative Ranks	1	.00	1.00
			Positive Ranks	1		
			Ties	96		
**13. When are you most active online on a normal weekend/ non-workday? (regular daytime/ evening use)**	97	95				
			Negative Ranks	1	-1.00	.32
			Positive Ranks	3		
			Ties	93		
**14. Have you ever used the internet to find information for a mental health, alcohol or substance use problem? (yes)**	61	74				
			Negative Ranks	2	-3.15	.002
			Positive Ranks	15		
			Ties	84		
**15. Do you think cyber-bullying is a serious problem for young people? (yes)**	93	81				
			Negative Ranks	11	-2.32	.02
			Positive Ranks	0		
			Ties	86		

**16i. In the past 12-months, how often have you been cyber-bullied? (not bullied)**	81	82				
			Negative Ranks	10	-.94	.35
			Positive Ranks	5		
			Ties	86		
**16ii. In the past 12 months, how often have you cyber-bullied someone? (never bullied)**	83	87				
			Negative Ranks	10	-1.41	.16
			Positive Ranks	5		
			Ties	84		
**17. Do you think sexting is a serious problem for young people your age? (yes)**	63	57				
			Negative Ranks	7	-1.67	.10
			Positive Ranks	2		
			Ties	86		
**18. In the past 12 months, have you had any of these things happen to on your mobile, smart phone or the Internet…**						
i. You have been sent a sexual message (yes)	46	51				
			Negative Ranks	3	-1.70	.08
			Positive Ranks	9		
			Ties	89		
ii. You have seen a sexual message posted where other people could see it (yes)	30	27				
			Negative Ranks	11	-.69	.49
			Positive Ranks	8		
			Ties	82		
iii. You have been asked to talk about	35	36				

acts of a sexual nature with someone (yes)						
			Negative Ranks	9	-.23	.82
			Positive Ranks	10		
			Ties	82		
iv. You have been asked for a photo or video clip showing yourself nude or nearly nude (yes)	22	29				
			Negative Ranks	2	-2.11	.04
			Positive Ranks	9		
			Ties	90		
v. You have seen other people perform acts of a sexual nature (yes)	24	35				
			Negative Ranks	4	-2.52	.01
			Positive Ranks	15		
			Ties	82		
vi. None (yes)	28	32				
			Negative Ranks	4	-1.16	.25
			Positive Ranks	8		
			Ties	89		
vii. Don't know (yes)	0	2				
			Negative Ranks	0	-1.41	.16
			Positive Ranks	2		
			Ties	99		
viii. I would prefer not to respond (yes)	0	1				
			Negative Ranks	0	-1.00	.32
			Positive Ranks	1		
			Ties	100		
**19. In the past 12 months, have you done these things on your mobile, smart phone or the Internet…**						
i. Sent someone a sexual message (yes)	30	41				
			Negative Ranks	0	-3.32	.001
			Positive Ranks	11		
			Ties	90		
ii. Posted a sexual message where other	1	5				

people could see it (yes)						
			Negative Ranks	0	-2.00	.05
			Positive Ranks	4		
			Ties	97		
iii. Talked about acts of a sexual nature with someone (yes)	33	42				
			Negative Ranks	5	-2.07	.04
			Positive Ranks	14		
			Ties	82		
iv. Asked someone to send you a photo or video clip showing themselves nude or nearly nude (yes)	9	13				
			Negative Ranks	2	-1.41	.16
			Positive Ranks	6		
			Ties	93		
v. Sent someone a photo or video of yourself nude or nearly nude (yes)	15	23				
			Negative Ranks	3	-2.14	.03
			Positive Ranks	11		
			Ties	87		
vi. None (yes)	51	48				
			Negative Ranks	8	-.83	.41
			Positive Ranks	5		
			Ties	88		
vii. Don't know (yes)	0	2				
			Negative Ranks	0	-1.41	.16
			Positive Ranks	2		
			Ties	99		
viii. I would prefer not to respond (yes)	0	2				
			Negative Ranks	0	-1.41	.16
			Positive Ranks	2		
			Ties	99		
**2. Do any of the following issues concern you personally…**						
i. Alcohol (yes)	19	17				

			Negative Ranks	9	-.50	.62
			Positive Ranks	7		
			Ties	82		
ii. Body Image (yes)	35	65				
			Negative Ranks	3	-4.27	<.001
			Positive Ranks	26		
			Ties	68		
iii. Bullying or emotional abuse (yes)	26	27				
			Negative Ranks	10	-.22	.83
			Positive Ranks	11		
			Ties	77		
iv. Coping with stress (yes)	65	68				
			Negative Ranks	11	-.96	.34
			Positive Ranks	16		
			Ties	70		
v. Depression (yes)	37	44				
			Negative Ranks	7	-1.50	.13
			Positive Ranks	14		
			Ties	76		
vi. Drugs (yes)	2	1				
			Negative Ranks	5	.33	.74
			Positive Ranks	4		
			Ties	90		
vii. Self-harm (yes)	11	12				
			Negative Ranks	3	-.71	.48
			Positive Ranks	5		
			Ties	89		

a. unless otherwise stated
b. mean age (standard deviation)
