# Supplementary material for: Comparison of Self-Reported Telephone Interviewing and Web-Based Survey Responses: Findings From the Second Australian Young and Well National Survey
Source: JMIR Ment Health. 2017 Sep 26;4(3):e37. doi: 10.2196/mental.8222 (PMC5635234; doi:10.2196/mental.8222)
Supplement: Multimedia Appendix 2 [file mental_v4i3e37_app2.pdf]

Multimedia Appendix 2.

Second Australian Young and Well National Survey 2014 results and sub-study results

	National Survey 2014		Substudy 2014	
	CATI	Online	CATI	Online
**Questionnaire item**	**Item endorsement (%[a])**	**Item endorsement (%[a])**	**Item endorsement (%[a])**	**Item endorsement (%[a])**
N	1400	1496	101	101
**1. Sex (female)**	49.6	63.9	60.4	60.4
**2. Age[b]**	20.2 (2.7)	19.1 (3.0)	20.2 (2.9)	20.2 (2.9)
**3. EET (yes)**	92.0	90.1	95.0	82.2
**4. Highest level of education (tertiary)**	48.3	35.8	53.5	51.5
**5. How would you rate your overall mental health in the past four weeks?**				
very good	31.4	17.8	28.7	23.8
good	39.5	28.5	37.6	39.6
moderate	21.7	32.4	26.7	26.7
bad	6.2	16.1	4.0	7.9
very bad	1.2	5.3	3.0	2.0
**6. Have you ever been diagnosed with a mental health or behavioural problem? (yes)**	23.9	31.6	25.0	31.6
**7i. In the past 12 months have you ever felt that life is hardly worth living? (yes)**	21.3	43.9	23.0	27.4
**7ii. In the past 12 months have you ever thought about taking your own life? (yes)**	15.4	33.5	11.1	18.4
**8. How long do you think a mental health or behavioural problem needs to be present before a young person should seek help? (< 4 weeks)**	70.5	63.0	66.3	72.0
**9. Would you know where to get help if you, or someone you knew, was feeling suicidal? (agree/ strongly agree)**	62.9	67.3	82.0	80.8

**1. Do you use the internet? (yes)**	100.0	98.9	100.0	100.0
**11. How often do you use the Internet? (everyday/ almost everyday)**	99.2	99.4	100.0	100.0
**12. When are you most active online on a normal weekday/ workday? (regular daytime/ evening use)**	94.9	96.0	97.0	97.0
**13. When are you most active online on a normal weekend/ non-workday? (regular daytime/ evening use)**	94.8	93.6	97.0	94.9
**14. Have you ever used the internet to find information for a mental health, alcohol or substance use problem? (yes)**	50.5	71.9	61.4	74.3
**15. Do you think cyberbullying is a serious problem for young people? (yes)**	94.8	73.9	93.1	80.6
**16i. In the past 12-months, how often have you been cyberbullied? (not bullied)**	83.6	74.2	81.2	82.2
**16ii. In the past 12 months, how often have you cyberbullied someone? (never bullied)**	82.0	81.7	83.2	86.9
**17. Do you think sexting is a serious problem for young people your age? (yes)**	55.8	54.7	62.8	57.1
**18. In the past 12 months, have you had any of these things happen to on your mobile, smart phone or the Internet…**				
i. You have been sent a sexual message (yes)	46.0	19.7	45.5	51.5
ii. You have seen a sexual message posted where other people could see it (yes)	42.7	10.8	29.7	26.7
iii. You have been asked to talk about acts of a sexual nature with someone (yes)	34.9	13.3	34.7	35.6
iv. You have been asked for a photo or video clip showing yourself nude or nearly nude (yes)	31.1	12.8	21.8	28.7
v. You have seen other people perform acts of a sexual nature (yes)	38.6	11.8	23.8	34.7
vi. None (yes)	26.9	16.6	27.7	31.7

vii. Don't know (yes)	.0	.8	.0	2.0
viii. I would prefer not to respond (yes)	.3	1.6	.0	1.0
**19. In the past 12 months, have you done these things on your mobile, smart phone or the Internet…**				
i. Sent someone a sexual message (yes)	32.1	14.8	29.9	40.6
ii. Posted a sexual message where other people could see it (yes)	1.7	1.3	1.0	5.0
iii. Talked about acts of a sexual nature with someone (yes)	36.6	14.6	32.7	41.6
iv. Asked someone to send you a photo or video clip showing themselves nude or nearly nude (yes)	12.1	4.8	8.9	12.9
v. Sent someone a photo or video of yourself nude or nearly nude (yes)	16.9	9.5	14.9	22.8
vi. None (yes)	56.4	24.3	50.5	47.8
vii. Don't know (yes)	.0	.7	.0	2.0
viii. I would prefer not to respond (yes)	.6	1.7	.0	2.0
**2. Do any of the following issues concern you personally…**				
i. Alcohol (yes)	18.9	14.6	19.0	17.2
ii. Body Image (yes)	47.7	74.8	34.7	65.3
iii. Bullying or emotional abuse (yes)	29.1	27.1	25.7	26.5
iv. Coping with stress (yes)	65.0	81.5	65.0	68.4
v. Depression (yes)	35.7	54.8	37.0	43.9
vi. Drugs (yes)	16.2	10.9	10.9	10.1
vii. Self-harm (yes)	15.5	23.6	10.9	12.4

a. Unless otherwise stated

b. mean age (standard deviation)
